# The Dynamic Ebbinghaus: motion dynamics greatly enhance the classic contextual size illusion

**DOI:** 10.3389/fnhum.2015.00077

**Published:** 2015-02-18

**Authors:** Ryan E. B. Mruczek, Christopher D. Blair, Lars Strother, Gideon P. Caplovitz

**Affiliations:** Department of Psychology, University of Nevada RenoReno, NV, USA

**Keywords:** size perception, form-motion interaction, size illusion, motion illusion, Ebbinghaus illusion, Titchener circles, size contrast

## Abstract

The Ebbinghaus illusion is a classic example of the influence of a contextual surround on the perceived size of an object. Here, we introduce a novel variant of this illusion called the Dynamic Ebbinghaus illusion in which the size and eccentricity of the surrounding inducers modulates dynamically over time. Under these conditions, the size of the central circle is perceived to change in opposition with the size of the inducers. Interestingly, this illusory effect is relatively weak when participants are fixating a stationary central target, less than half the magnitude of the classic static illusion. However, when the entire stimulus translates in space requiring a smooth pursuit eye movement to track the target, the illusory effect is greatly enhanced, almost twice the magnitude of the classic static illusion. A variety of manipulations including target motion, peripheral viewing, and smooth pursuit eye movements all lead to dramatic illusory effects, with the largest effect nearly four times the strength of the classic static illusion. We interpret these results in light of the fact that motion-related manipulations lead to uncertainty in the image size representation of the target, specifically due to added noise at the level of the retinal input. We propose that the neural circuits integrating visual cues for size perception, such as retinal image size, perceived distance, and various contextual factors, weight each cue according to the level of noise or uncertainty in their neural representation. Thus, more weight is given to the influence of contextual information in deriving perceived size in the presence of stimulus and eye motion. Biologically plausible models of size perception should be able to account for the reweighting of different visual cues under varying levels of certainty.

## Introduction

To accurately guide interactions with objects in the world, the visual system must construct the perceived size of an object from its retinal image size. A variety of contextual cues can bias this constructive process, such as physical and perceived distance (Emmert, 1881; Ponzo, 1911; Boring, 1940; Berryhill et al., 2009), an object's geometrical and textural properties (Lotze, 1852; Kundt, 1863; Helmholtz, 1867; Murray et al., 2006; Westheimer, 2008; Giora and Gori, 2010), knowledge of an object's typical size (Konkle and Oliva, 2012), and the relative size of different objects in a scene (Robinson, 1972; Coren and Girgus, 1978; Roberts et al., 2005) or the frame around an object (Kunnapas, 1955; Rock and Ebenholtz, 1959; Robinson, 1972; Brigell et al., 1977). These biases are revealed by many classic visual illusions in which the size of an object is misperceived. Understanding the conditions under which illusory percepts do and do not occur can help to guide and constrain biologically plausible models of the underlying neural mechanisms that support size perception.

**Figure 1:**
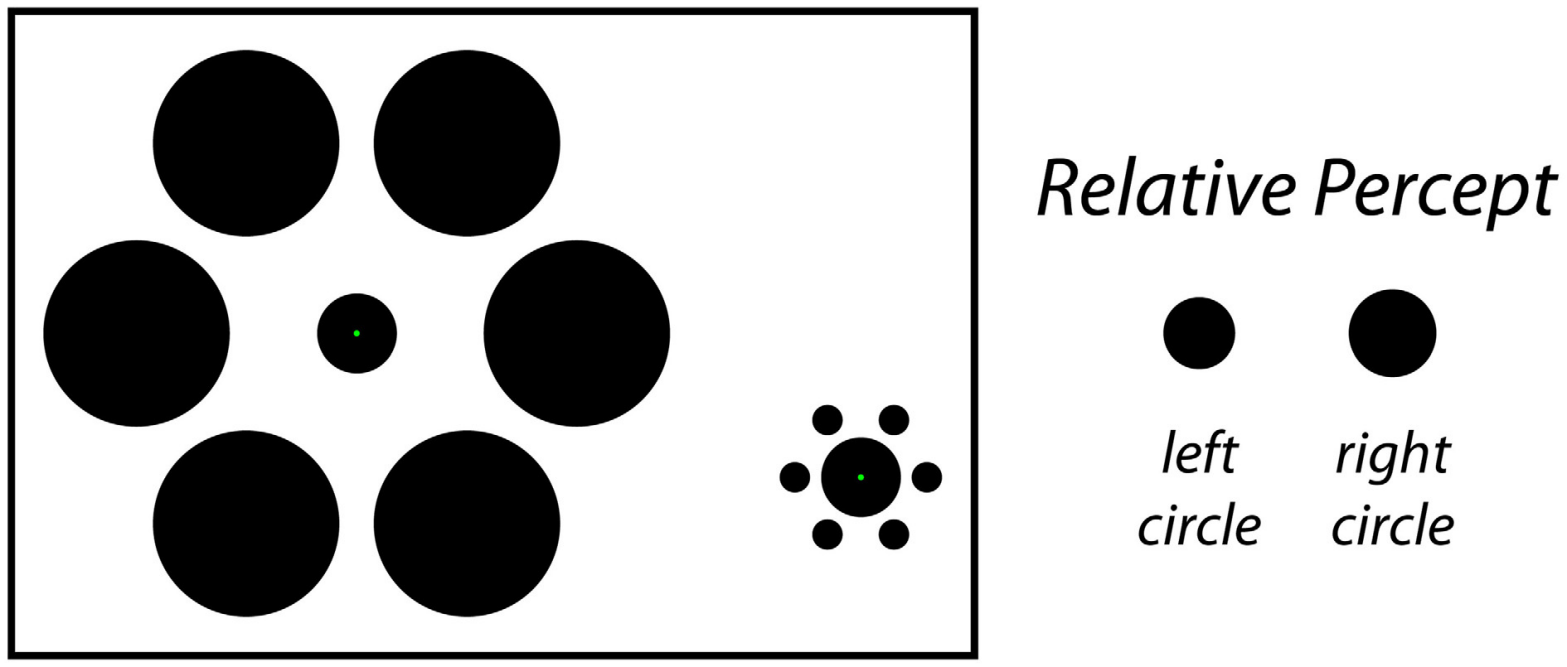
**The static Ebbinghaus illusion**. In this classic size-contrast illusion, the inner circle is perceived to be larger on the right and smaller on the left (right). In fact, both filled circles are the same physical size in the figure. The stimulus configuration depicted here matches the one used for the Static condition of Experiment 1, in which participants adjusted the physical size of the center circle on the left to perceptual match the size of the central circle on the right.

Classical size-contrast and size-assimilation illusions such as the Ebbinghaus illusion (Thiéry, 1896; Burton, 2001) or the Delboeuf illusion (Delboeuf, 1892; Nicolas, 1995) demonstrate that the presence of nearby objects can influence the perceived size of a central target object. In a typical demonstration of the Ebbinghaus illusion (Figure 1, left), a viewer is presented with two static stimulus configurations side-by-side. In the center of each is an identical target object surrounded by an array of similar objects. For one target, the surrounding objects are larger and farther away; for the other target, the surrounding objects are smaller and closer. Under these conditions, the viewer perceives the target that is surrounded by the large-and-far objects to be smaller than the target that is surrounded by the small-and-near objects (Figure 1, right), even though the two target objects are actually the same size. The magnitude of this effect is dependent on a variety of factors such as the size, number, eccentricity and spacing of the inducers (Morinaga, 1956; Oyama, 1962; Massaro and Anderson, 1971; Jaeger, 1978; Weintraub, 1979; Jaeger and Lorden, 1980; Weintraub and Schneck, 1986; Jaeger and Grasso, 1993; Ehrenstein and Hamada, 1995; Roberts et al., 2005), and the similarity between the target and inducers (Coren and Miller, 1974; Coren and Enns, 1993; Choplin and Medin, 1999). Based on previous reports, the classic Ebbinghaus illusion may cause a target circle to appear around 10–20% larger (or smaller) than it actually is (Massaro and Anderson, 1971; Jaeger and Pollack, 1977; Weintraub, 1979; Girgus and Coren, 1982; Roberts et al., 2005).

**Figure 2:**
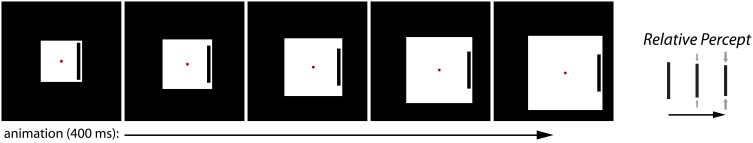
**The Dynamic Illusory Size Contrast (DISC) effect**. The black target bar is perceived to be shrinking when a surrounding white box grows (right). The effect requires both the relative size change between the surrounding box and the target bar, and additional stimulus dynamics resulting from eye movements (e.g., pursuit of the translating red fixation spot) or bar motion.

We recently introduced a novel illusion called the Dynamic Illusory Size-Contrast (DISC) effect, which highlights the role of dynamic visual information in modulating the contribution of different sources of information in determining the perceived size of an object (Mruczek et al., 2014). In the DISC effect, the viewer perceives the size of a target bar to be shrinking when (1) it is surrounded by an expanding box and (2) there are additional dynamic cues such as eyes movements or bar motion (Figure 2). Importantly, the expanding box is necessary but not sufficient to induce an illusory percept. Specifically, no illusory effect is observed when the size of the surrounding box is held constant but the eyes and bar move, or when the surrounding box expands but the eyes and bar remain stationary. Thus, the key factor in the DISC effect is the interaction between a size-contrast effect and additional stimulus motion. This requirement distinguishes the DISC effect from classic size-contrast illusions such as the Ebbinghaus illusion, which are purely static in nature. The DISC effect suggests that different cues (e.g., retinal image size and relative size) are weighted or integrated differently under certain viewing conditions. We previously proposed that the dynamic nature of the DISC stimulus leads to greater uncertainty regarding the image size of the target object because of motion-related noise at the level of the retinal input. As a result, other sources of information, such as relative size, contribute more to its perceived size, thereby greatly increasing the magnitude of the illusory percept (Mruczek et al., 2014). The information uncertainty hypothesis suggests that the DISC effect should not be limited to the specific stimulus we originally examined. Rather, we are proposing a general property of constructed size that should be observable in any stimulus configuration in which increased uncertainty in image size could lead to a re-weighting of contextual cues. In order to more fully evaluate the information uncertainty hypothesis, we present the Dynamic Ebbinghaus illusion, which incorporates the dynamic components of the DISC stimulus with the familiar concentric circles configuration of the Ebbinghaus stimulus. This allows us to quantify the illusory magnitude of the static and dynamic illusions using matched stimulus parameters and to confirm that the DISC effect is not limited to the specific stimulus configuration of our original experiment.

Our data reveal a set of surprising results, but ones that are consistent with the information uncertainty hypothesis and our interpretation of the DISC effect. What would a person perceive if they viewed a single, dynamic Ebbinghaus stimulus configuration, in which the size and eccentricity of the surrounding objects expanded and then contracted over time? If the inducers have a constant, automatic, and obligatory effect on perceived size, then the central target should appear to shrink and grow in opposition with the expansion and contraction of the surrounding objects. Here, we show that contrary to this intuitive prediction, the size of the central target appears to change relatively little (~7%); in this case, the illusory effect is less than half the measured magnitude of the classic, static illusion (~20%). However, if the entire stimulus translates across the screen requiring a smooth pursuit eye movement, the resulting illusory change in target size is dramatic (~36%); in this case, almost twice the magnitude of the classic, static illusion. Additionally, a particularly strong illusory effect can be obtained by combining a dynamic change in the inducers with a variety of motion sources: stimulus motion relative to fixation, smooth pursuit eye movements, and frame-by-frame jittering of the target position. A combination of these manipulations leads to the most striking illusion (~78%), one that is nearly four times the magnitude of the classic, static illusion.

The striking magnitude of the Dynamic Ebbinghaus illusion and the inherently dynamic nature of our visual environment suggest that the previously underappreciated influence of image dynamics is likely to play a role in everyday perception. Our data and hypothesis places constraints on the neural implementation of contextual influences, which are not automatic, but rather are reweighted depending on factors such as dynamic motion. Ultimately, biologically plausible models of size perception should be able to account for the reweighting of different visual cues under different levels of certainty.

## Materials and methods

### Participants

Twelve participants (4 female, ages 20–35) completed Experiment 1 and eighteen participants (9 female, ages 20–38) completed Experiment 2. Eleven participants, including two authors (R.E.B.M. and C.D.B.), completed both experiments. Data from two additional participants for Experiment 1 were excluded because they failed to correctly perform the task as instructed. Prior to participating, each observer provided informed written consent. All participants reported normal or corrected-to-normal vision and all participants, except the two authors, were naïve to the specific aims and designs of the experiments. All procedures were approved by the Institutional Review Board of the University of Nevada, Reno.

### Apparatus and display

Stimuli were created and presented with the Psychophysics Toolbox (Brainard, 1997; Pelli, 1997) for MATLAB (Mathworks Inc., Natick, MA) on one of two setups. Setup 1 used a ViewSonic Graphic Series G220fb monitor (20 in, 1024 × 768 pixel resolution, 85-Hz refresh rate) driven by a Mac Mini computer (2.5-GHz, 16 GB of DDR3 SDRAM) with an Intel HD Graphics 4000 graphics processor (768 MB). Setup 2 used a Dell Triniton P1130 monitor (20 in, 1024 × 768 pixel resolution, 85-Hz refresh rate) driven by a Mac Pro computer (2.4-GHz, 12 GB of DDR3 SDRAM) with an ATI Radeon HD 5870 graphics processor (1024 MB). For both setups, participants viewed the stimuli binocularly from a distance of 70 cm with their chin positioned in a chin-rest. The stimuli consisted of filled black (Setup 1: 0.44 cd/m^2^; Setup 2: 3.35 cd/m^2^) circles on a white (Setup 1: 100.20 cd/m^2^; Setup 2: 105.10 cd/m^2^) background, with a green fixation point (Setup 1: 65.37 cd/m^2^; Setup 2: 75.90 cd/m^2^). Eleven of the participants in each Experiment used Setup 1.

### Experiments goals, stimuli, and procedures

#### Experiment 1

***Goal of Experiment 1***. The goal of Experiment 1 was to determine whether distinct types of image dynamics would modulate the magnitude of the Ebbinghaus illusion. Specifically, we examined whether dynamically modulating the size and eccentricity of the inducers would lead to dynamic modulations in the perceived size of a fixated target (Movie 1). We also examined whether global motion of the entire stimulus (Movie 2) would influence the degree to which such dynamic modulation is observed (as is the case for the original DISC effect).

According to the information uncertainty hypothesis, we would predict that simply modulating the size and eccentricity of the inducing stimuli should have little to no effect on the perceived size of the target. This is because the projected image of the target stimulus should be largely stationary and constant throughout the duration of a trial, thereby providing a strong and high quality signal that the size of the target is not changing, despite the modulations in the contextual circles. In contrast, when the entire stimulus is put into motion, participants must execute eye movements to track the target. Despite effort to maintain fixation, imperfect pursuit gain and catch-up saccades will lead to a highly dynamic and non-stationary representation of the target, thereby providing an uncertain signal as to the size of the target. In this instance, the hypothesis predicts that a greater emphasis will be placed on the contextual cues provided by the modulating inducers leading to a robust modulation in the perceived size of the target.

In Experiment 1 we also compared the magnitude of the dynamic illusions with the magnitude of the classic, static illusion (Figure 1) using matched stimulus parameters. This provides a baseline for comparing the effects of image dynamics on the perceived size of the target. Finally, we included control conditions in which no inducers were present. This allowed us to quantify the magnitudes of the inducer-related illusion in isolation from any response bias or other non-contextual factors that may influence the perceived size of the target.

**Figure 3:**
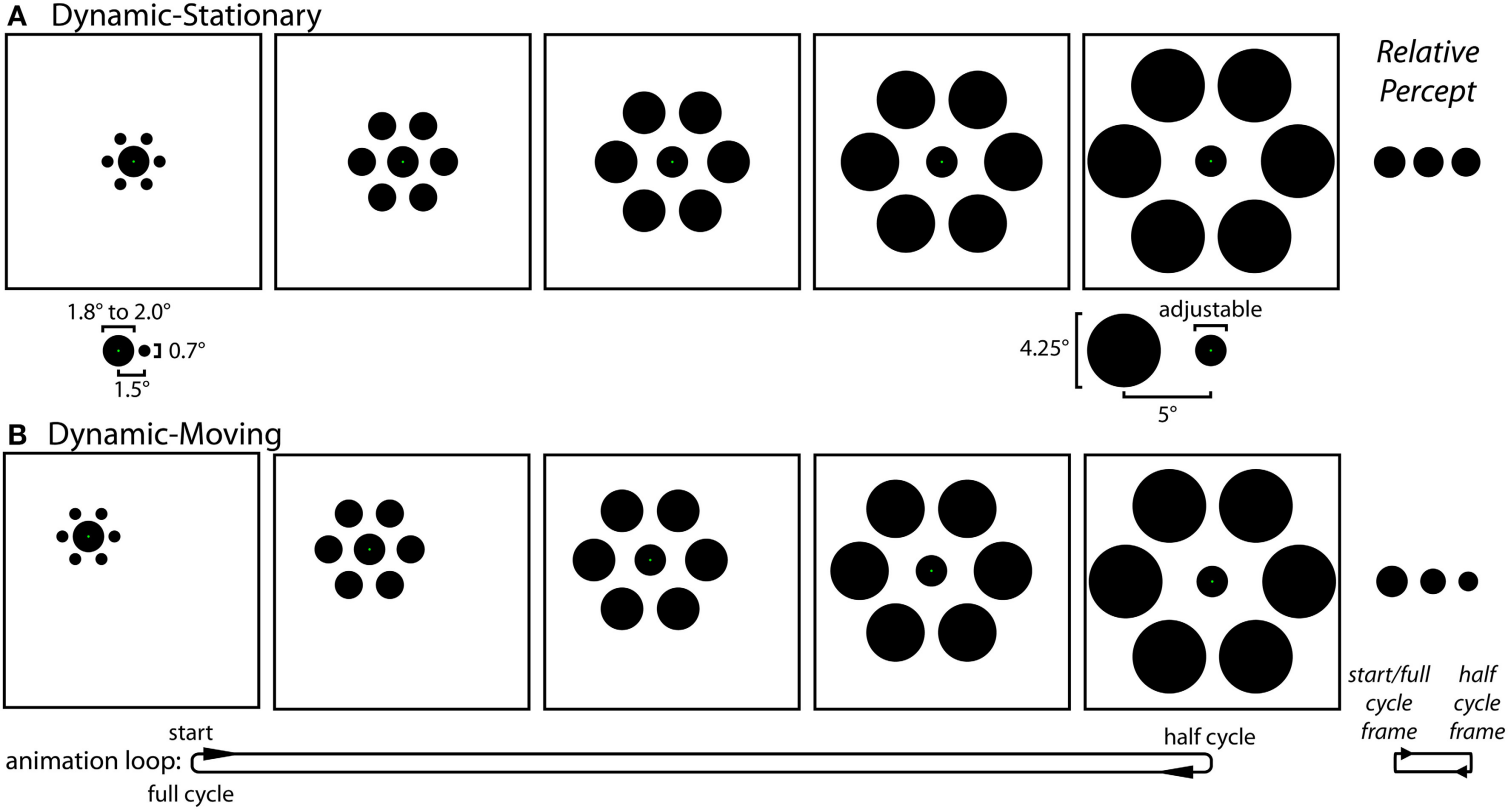
**The Dynamic conditions of Experiment 1**. **(A)** In the Dynamic-Stationary condition (Movie 1) the inducers continuously modulate their size and eccentricity between small-and-near and large-and-far while the participant fixates a stationary central target. The relative size and eccentricity of the target and the small-and-near (left) and large-and-far (right) inducers used in all conditions of both experiments are shown below their respective frames. **(B)** In the Dynamic-Moving condition (Movie 2) there is an additional translation of the entire stimulus across the screen, requiring participants to track the green fixation spot with a smooth pursuit eye movement. Although participants adjusted the rate of change in the size of the central circle (by adjusting its size at the half-cycle point) during the experiment, these panels depict the situation in which the target does not actually change size. The effect of motion on perceived size of the central circle is relatively weak for the Dynamic-Stationary condition and much stronger in the Dynamic-Moving condition (right).

***Experiment 1 stimuli and procedures***. Experiment 1 contained three distinct experimental conditions: Static (Figure 1), Dynamic-Stationary (Figure 3A and Movie 1), and Dynamic-Moving (Figure 3B and Movie 2). For all three conditions, participants saw an equal number of trials with and without inducers. The experiment used a self-adjustment technique in which the participant adjusted a physical property of a target circle (diameter for the Static condition and growth rate for the two Dynamic conditions) using a computer mouse in order to match the size of two circles (Static condition) or to minimize the perceived change in size over time (Dynamic conditions). Throughout all trials, participants were instructed to maintain fixation on a green fixation spot (0.1° width), the exact location of which depended on the particular condition (described below).

*Static condition (Figure 1)*. The Static condition was the familiar and classic Ebbinghaus illusion. In this condition, participants viewed two Ebbinghaus configurations side-by-side, each formed by a central circle surrounded by six equally spaced inducers. The center of the left target circle was positioned 6° to the left and 2° above the center of the monitor and the center of the right standard circle was positioned 6° to the right and 1° below the center of the monitor, each with an additional positional jitter of 1° at a random angle on a given trial. On each Static trial, one stimulus contained large-and-far inducers (4.25° diameter, 5° eccentricity) and the other contained small-and-close inducers (0.7° diameter, 1.5° eccentricity; Figure 3A, bottom), with half of the trials having large-and-far inducers on the left (i.e., surrounding the adjustable target) and half containing the large-and-far inducers on the right (i.e., surrounding the non-adjustable standard). The diameter of the standard circle in the right stimulus configuration was either 1.8° or 2.0°. Participants adjusted the size of the target circle in the center of the left stimulus using a computer mouse until the size of the central circles in the right and left stimulus configurations were perceptually equivalent. The target circle could be adjusted between 0.2° and a diameter that would just touch (but never overlap with) the inducers (2.3° or 5.75° when the target was surrounded by small-and-close or large-and-far inducers). The size of the target at the start of each trial was set to within 10% of one extreme, with half of the trials starting from the smallest extreme and half starting from the largest extreme. For all trials, a change in the mouse position of 1 pixel, corresponding to the finest resolution of control, was equivalent to a change in target width of 0.0072° (~0.004% of the standard circle). For the Static condition, green fixation points were located on target and standard circles and participants were instructed to move their gaze between these two fixation points when matching the circle sizes. In a given session, there were a total of 24 Static condition trials with inducers (2 standard diameters × 2 initial target sizes × 2 size of inducers surrounding the adjustable target × 3 repetitions). In addition, there were 24 corresponding without-inducer trials in which everything remained the same except the inducers were not presented (2 standard diameters × 2 initial target sizes × 6 repetitions). In these cases, participants matched the sizes of two isolated circles.

*Dynamic-Stationary condition (Figure 3A and Movie 1)*. For the Dynamic-Stationary condition, participants viewed a single Ebbinghaus configuration in which the size and eccentricity of the six equally spaced inducers changed smoothly over time between small-and-close (0.7° diameter, 1.5° eccentricity) and large-and-far inducers (4.25° diameter, 5° eccentricity; Figure 3A, bottom). The size and relative positions of the stimulus elements were matched to those in the Static condition in all respects. On half of the Dynamic-Stationary trials, the inducers initially expanded, with a full cycle of the animation comprised of the inducers expanding from small-and-close to large-and-far and then contracting back to small-and-close again. On the other half of trials the inducers initially contracted, with a full cycle of the animation comprised of the inducers contracting from large-and-far to small-and-close and then expanding back to large-and-far. A full cycle of the animation covered a duration of 1.4 s, at the end of which the animation was immediately repeated, leading to a continuous looping of the dynamic change in inducer size and eccentricity. In addition to the inducers, the central target circle also changed size smoothly over time, growing or shrinking over the first half of a cycle, and then transitioning back to its initial (standard) size over the second half of the cycle. The animation cycle was continuously repeated as the participant adjusted the growth rate of the target using a computer mouse until there was no perceptible change in the size of the target over the course of the animation. The growth rate was altered by adjusting the final size of the target circle half way through the animation cycle, with the circle always starting from a standard width of 1.8 or 2.0°. The target growth rate could be adjusted such that extremes represented a target that shrank to 0.2° or grew to the point that it would just touch (but never overlap with) the inducers (2.3 or 5.75° when the target was surrounded by small-and-close or large-and-far inducers half way through the animation cycle). The growth rate at the start of each trial was set to within 10% of one extreme, with half of the trials starting from the smallest extreme and half starting from the largest extreme. For all trials, a change in the mouse position of 1 pixel, corresponding to the finest resolution of control, was equivalent to a change in target growth rate of 0.0103°/s (0.0072°, or ~0.004% of the standard circle, over the 700 ms half-cycle of the animation period). The green fixation point was located at the center of the target circle and participants were instructed to maintain fixation on this point throughout the trial. In a given session, there were a total of 24 Dynamic-Stationary condition trials with inducers (2 starting standard diameters × 2 directions of inducer modulation × 2 initial target growth rates × 3 repetitions). In addition, there were 24 corresponding without-inducer trials in which everything remained the same except the inducers were not presented (2 starting standard diameters × 2 initial target growth rates × 6 repetitions). In these cases, participants minimized the perceived modulation of size for a single, stationary isolated circle.

*Dynamic-Moving condition (Figure 3B and Movie 2)*. The Dynamic-Moving condition was similar to the Dynamic-Stationary condition in all respects, except the entire stimulus configuration translated across the screen over the animation period. Over the first half of a given cycle, the stimulus translated down and to the right at a 45° angle over a distance of 3.5° of visual angle, which corresponds to a rate of 5°/s. During the second half of the cycle, the stimulus translated back to its initial starting position. As was previously the case, the cycles would continuously repeat as the participant adjusted the growth rate of the target. The green fixation point was again located at the center of the target circle and participants were instructed to track this point throughout the trial. Note that with the exception of differences that arise due to imperfect smooth pursuit, the retinal stimulation was perfectly matched across the Dynamic-Stationary and Dynamic-Moving conditions. In a given session, there were a total of 24 Dynamic-Moving condition trials with inducers (2 starting standard diameters × 2 directions of inducer modulation × 2 initial target growth rates × 3 repetitions). In addition, there were 24 corresponding without-inducer trials in which everything remained the same except the inducers were not presented (2 starting standard diameters × 2 initial target growth rates × 6 repetitions). In these cases, participants minimized the perceived modulation of size for a single, moving isolated circle.

Altogether, each participant completed a total of 144 pseudorandomly ordered trials in a single session. There was no limit on the amount of time participants had to respond on a given trial and participants did not receive any feedback on the accuracy of their responses for any trial. Eye movements were not monitored and the cursor was not visible to the participants at any point during the experiment.

#### Experiment 2

***Goal of Experiment 2***. The goal of Experiment 2 was to further explore how distinct types of image dynamics, particularly those that arise due to eye movements and stimulus motion, may influence the magnitude of the Dynamic Ebbinghaus illusion. There are multiple ways in which motion can be added to the stimulus, and by extension uncertainty added to the image size representation of the target. Experiment 2 was designed to determine the degree to which different sources of stimulus motion contribute to the magnitude of the Dynamic Ebbinghaus illusion. The experiment contained six distinct dynamic conditions defined by the location of fixation and whether or not the eyes moved, the target was translating, or the target position was jittered.

***Experiment 2 stimuli and procedures***. Aside from the following three differences, the basic sizes, positions and timings of the animated stimuli were the same as in Experiment 1. First, on trials in which the entire stimulus translated, the direction of translation was 60° from horizontal (instead of 45°) so that the upper-left inducer was stationary over the course of the animation period. Second, Experiment 2 only included trials in which the inducers initially expanded and then contracted. Third, the starting (standard) diameter of the target circle was randomly selected from a uniform distribution between 1.8° and 2.0° on each trial. As stated above, there were six distinct stimulus conditions, each with a matched without-inducer control condition. In order to keep track of the different conditions, each one was assigned a distinct descriptive name.

*Stationary condition*. The Stationary condition was exactly the same as the Dynamic-Stationary condition of Experiment 1 (Figure 3A and Movie 1), with participants fixating the stationary target circle. As in all conditions of Experiment 2, the inducers dynamically modulated between small-and-near (0.7° diameter, 1.5° eccentricity) and large-and-far (4.25° diameter, 5° eccentricity, Figure 3A, bottom).

**Figure 4:**
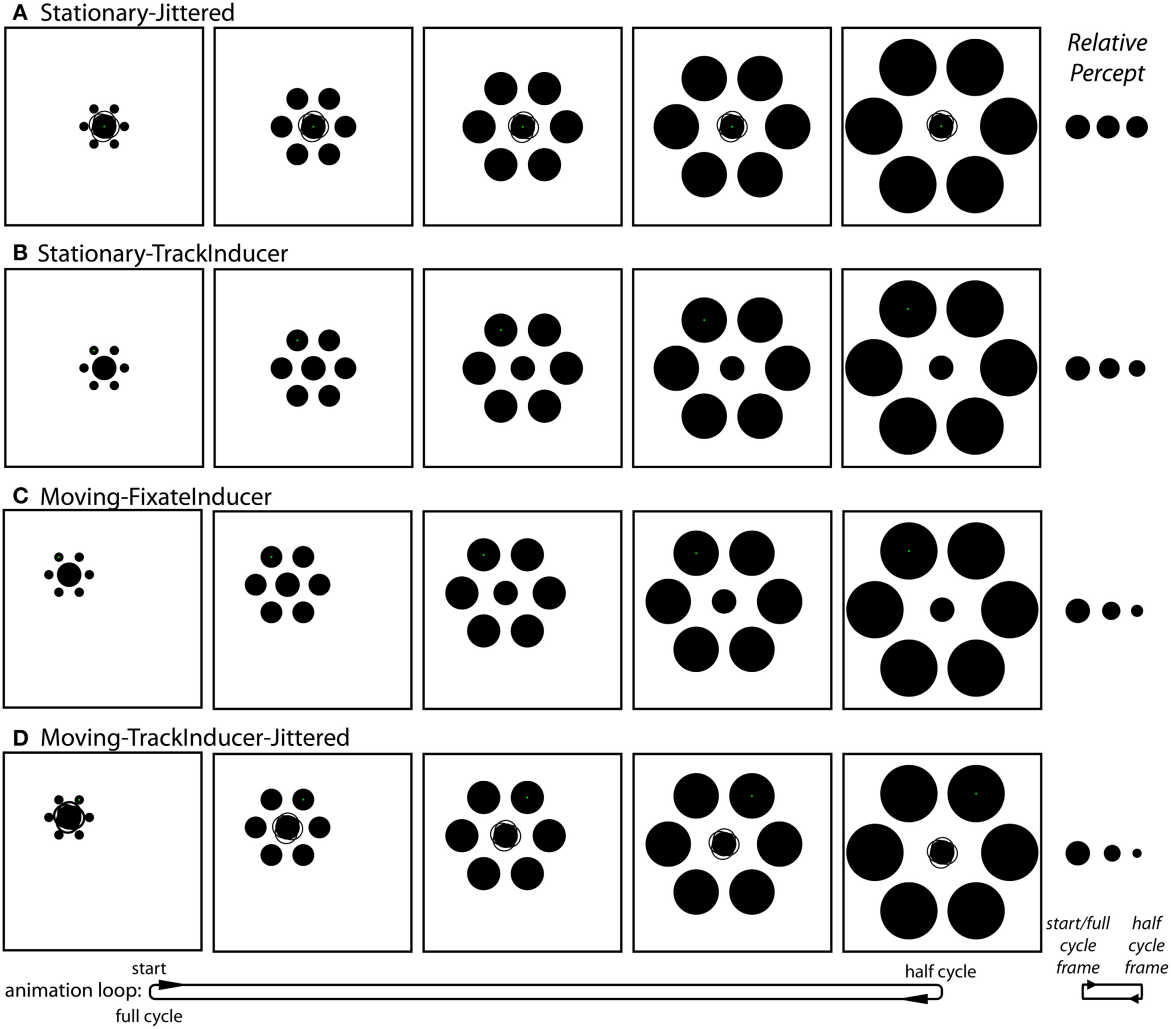
**The conditions unique to Experiment 2**. **(A)** The Stationary-Jittered condition (Movie 3). Jittering of the target position is depicted by the faint partial circle outlines. **(B)** The Stationary-TrackInducer condition (Movie 4). Participants tracked the upper left inducer with a smooth pursuit eye movement. **(C)** The Moving-FixateInducer condition (Movie 5). Participants fixated the upper left inducer, which was stationary. **(D)** The Moving-TrackInducer-Jittered condition (Movie 6). This condition contained all of the manipulations that were isolated in other conditions: smooth pursuit, peripheral viewing, a change in target eccentricity, and frame-by-frame jittering of the target position. Although participants adjusted the rate of change in the size of the central circle (by adjusting its size at the half-cycle point) during the experiment, these panels depict the situation in which the target does not actually change size. As can be seen in the corresponding demo movies, the strongest effects (right) are observed in the Moving-FixateInducer and Moving-TrackInducer-Jittered conditions.

*Stationary-Jittered condition (Figure 4A and Movie 3)*. The Stationary-Jittered condition was similar to the Stationary condition with an additional random jitter in the position of central target circle. On each frame of the animation, the position of the target was offset by 0.06° (~2 pixels) in a random direction from the center of the stimulus configuration. This manipulation served to artificially increase the uncertainty in the position of the target integrated over time.

*Stationary-TrackInducer condition (Figure 4B and Movie 4)*. The Stationary-TrackInducer condition was similar to the Stationary condition with the exception that the green fixation point was positioned such that it was centered on the upper-left inducer, which itself translated over time between 1.5° and 5° eccentricity as the inducers dynamically changed from small-and-near to large-and-far. Thus, participants were required to track the moving fixation point over time, which led to a change in the eccentricity of the peripherally positioned target circle.

*Moving condition*. The Moving condition was essentially the same as the Dynamic-Moving condition of Experiment 1 (Figure 3B and Movie 2), except for the angle of stimulus translation. Participants fixated the central target circle while the entire stimulus translated down and to the right at a 60° angle over a distance of 3.5°, which corresponds to a rate of 5°/s. As with the Dynamic conditions of Experiment 1, the Stationary and Moving conditions of Experiment 2 were largely matched for retinal stimulation.

*Moving-FixateInducer condition (Figure 4C and Movie 5)*. The Moving-FixateInducer condition was similar to the Moving condition with the exception that the green fixation point was centered on the upper-left inducer, which itself was stationary over the animation period. Thus, participants were required to maintain fixation and covertly attend to a peripheral target that itself changed eccentricity between 1.5° and 5° over time. Aside from differences arising from imperfect smooth pursuit, the retinal stimulation was perfectly matched across the Moving-FixateInducer and Stationary-TrackInducer conditions.

*Moving-TrackInducer-Jittered condition (Figure 4D and Movie 6)*. The Moving-TrackInducer-Jittered condition was similar to the Moving and Moving-FixateInducer conditions with the following exceptions. First, the green fixation point was centered on the upper-right inducer, which itself translated to the right 3.5° over the first half of the animation period, and then back to its original position. Thus, participants were required to track a moving inducer over time. In addition, the relative eccentricity of the peripherally positioned target circle changed between 1.5° and 5°. Finally, on each frame of the animation, the position of the target was offset by 0.06° (~2 pixels) in a random direction from the center of the stimulus configuration. Thus, the Moving-TrackInducer-Jittered condition combined many of the manipulations tested in isolation by the other conditions: peripheral viewing and changes in target eccentricity, smooth pursuit eye movements, and target position jitter.

In a given session, there were a total of 12 trials for each of the six conditions with inducers (2 initial target growth rates × 6 repetitions). In addition, there were 12 corresponding without-inducer trials for each of the six conditions in which everything remained the same except the inducers were not presented (2 initial target growth rates × 6 repetitions). In total, each participant completed 144 pseudorandomly ordered trials in a single session.

### Quantifying the magnitude of the dynamic ebbinghaus illusion

#### Point of subjective equality (PSE)

The response on each trial provided an estimate of the point of subjective equality (PSE) for that condition. For the Dynamic conditions in both Experiments, the PSE represents the size of the target circle half way through the animation cycle (which defines the growth rate of the target) such that the participant perceived a minimal change in the size of the central circle over the entire animation (e.g., subjectively equivalent to an unchanging circle). For the Static condition of Experiment 1, the PSE represents the size of the target circle such that the participant perceived the target and standard circles to be the same size.

PSEs are reported as a percentage increase (positive) or decrease (negative) from the starting circle (i.e., standard) size for dynamic trials or from the standard size for static trials. In all cases the PSEs were calculated separately for trials with and without inducers. To account for potential outliers, we took the conservative approach of discarding, for each participant, the trials with the highest and lowest PSE for each condition, separately for the with- and without-inducer trials.

#### Illusion magnitude

The final metric of the illusion magnitude for a given condition was calculated as the difference between the mean PSEs for trials with inducers and trials without inducers. This allowed us to account for potential response biases and to isolate the effects of the dynamic inducers from the stimulus dynamics of the target circle and the eyes. For example, objects viewed in the periphery may be perceived as smaller compared to the same object viewed at the fovea (Helmholtz, 1867; James, 1890; Bedell and Johnson, 1984). In conditions in which participants covertly attended to a peripheral target that changed in eccentricity over time (e.g., Stationary-TrackInducer condition of Experiment 2), the fact that the target changed eccentricity may lead to a perceived change in the size of the target. This will be apparent regardless of whether or not there are inducers present. Thus, although some significant non-inducer-specific biases were observed (see Supplementary Figure 1A for data and discussion), they do not contribute to the final illusion magnitudes reported below.

To equate the sign for illusion magnitudes derived from trials in which the inducers initially expanded or initially contracted (for dynamic conditions), which are predicted to have opposite effects on the perceived size of the target (see Supplementary Figure 1A), we inverted the sign of the illusion magnitude for trials in which the inducers initially shrank. A similar sign inversion was applied to illusion magnitudes derived from the Static condition of Experiment 1 for trials in which the adjusted target was surrounded by small-and-near inducers.

If there were no illusory effect for a given condition (i.e., veridical perception), we would anticipate an illusion magnitude of zero. For the Dynamic conditions, an illusion magnitude greater than zero indicates that the target had to physically change in size over the animation period in the same direction as the inducers (e.g., a physically growing target when the inducers grew over the first half of the animation period) in order to be perceived as unchanging. For the Static condition, an illusion magnitude greater than zero indicates that the physical size of the target had to be more like the surrounding inducers compared to the standard (e.g., a physically larger target when surrounded by large inducers).

#### Statistical analyses

To avoid the assumptions of parametric statistical tests, we analyzed the data using a series of standard non-parametric tests and randomization procedures. However, we note that when the data were analyzed using parametric alternatives, the significance and the interpretation of the results were not qualitatively different (see Supplementary Material). To determine whether there was an illusory percept observed for a given condition, illusion magnitudes were compared against zero using a non-parametric Wilcoxon signed-rank test. To determine whether the experimental manipulations differentially influenced the illusion magnitude, pair-wise comparisons across conditions within an experiment were performed using a two-tailed non-parametric permutation test for paired data. The mean difference in illusion magnitude across two conditions was compared to a distribution of differences obtained for every possible permutation of each participant's values (number of permutations = 2^*N*^, where *N* is the number of participants for that Experiment; *n*_*perm*_ = 2^12^ = 4,096 for Experiment 1; *n_perm_* = 2^18^ = 262,144 for Experiment 2). This is equivalent to randomly flipping the sign of the illusion magnitude difference across the two conditions for each participant. For this test, the *p*-value was defined as the proportion of random permutations of the data that yielded a difference in the mean illusion magnitudes for two conditions that was equal to or greater than the actual observed difference. The paired comparisons followed an initial non-parametric Friedman's test for repeated-measures data to verify a main effect of condition. As we emphasize only a small number of all possible comparisons informed by our experimental design and initial hypotheses, we report uncorrected *p*-values and assess statistical significance using an α of 0.05.

## Results

### Results of experiment 1

**Figure 5:**
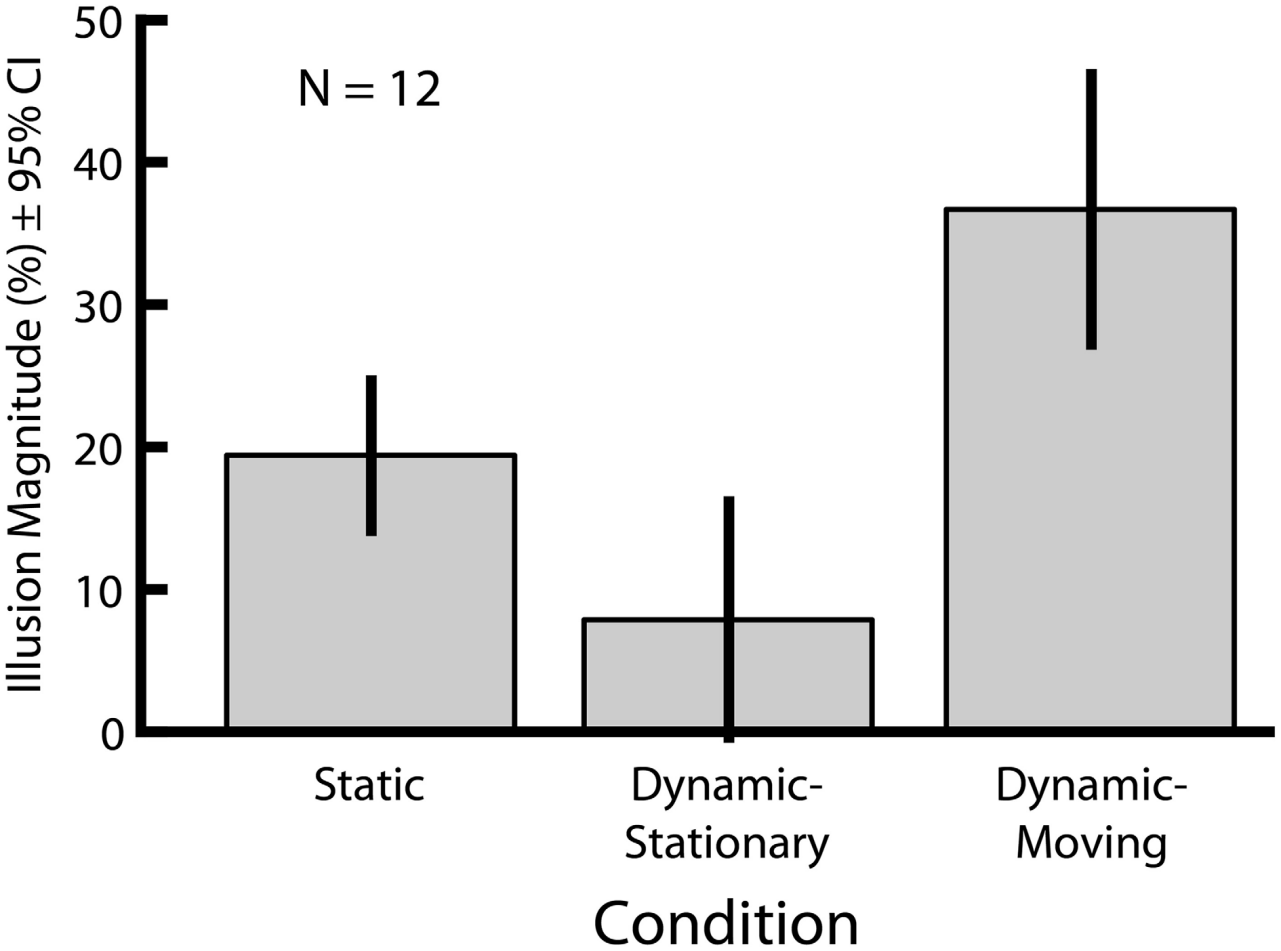
**Mean illusion magnitudes for Experiment 1**. All three conditions lead to an illusory modulation of perceived size (*p* = 0.0005 in all cases, one-sample Wicoxon signed-rank test). All pairwise comparisons revealed significant differences in illusion magnitudes across conditions (*p* ≤ 0.027 in all cases, permutation test). Error bars represent 95% confidence intervals.

In Experiment 1 we directly compared the magnitudes of two versions of the Dynamic Ebbinghaus illusion (Figure 3 and Movies 1, 2) with the classic static Ebbinghaus illusion (Figure 1) using a set of matched stimulus parameters (Figure 3A, bottom). Figure 5 shows the mean illusion magnitudes across participants, which were significantly greater than zero for all three conditions (*p* = 0.0005 in all cases, one-sample Wilcoxon signed-rank test). For the Static condition, the positive illusion magnitudes reflect the expected Ebbinghaus illusion: the physical size of the adjustable target had to be biased in the direction of the surrounding inducers to be perceived as being the same size as the standard. For the Dynamic conditions, the positive illusion magnitudes are again consistent with the expected effect: the center circle had to be physically changing size in the same direction as the inducers for participants to perceive it as not changing over the course of the animation. Friedman's test revealed a highly significant difference among the distributions of the illusion magnitudes across the three conditions [χ^2^_(2)_ = 20.17, *p* = 0.0001]. Illusion magnitudes were highest for the Dynamic-Moving condition (*M* = 37.3%, *SE* = 4.7%), significantly higher than both the Static condition (*M* = 19.9%, *SE* = 2.4%; *p* = 0.0024, permutation test) and the Dynamic-Stationary condition (*M* = 8.1%, *SE* = 3.7%, *p* = 0.0005, permutation test). In addition, illusion magnitudes for the Dynamic-Stationary condition were significantly lower than those for the Static condition (*p* = 0.024, permutation test). Thus, the magnitude of the Dynamic-Moving (Movie 2) illusion was almost twice that of the classic static illusion and the magnitude of the Dynamic-Stationary (Movie 1) illusion was less than half that of the classic Static illusion (Figure 1).

### Results of experiment 2

**Figure 6:**
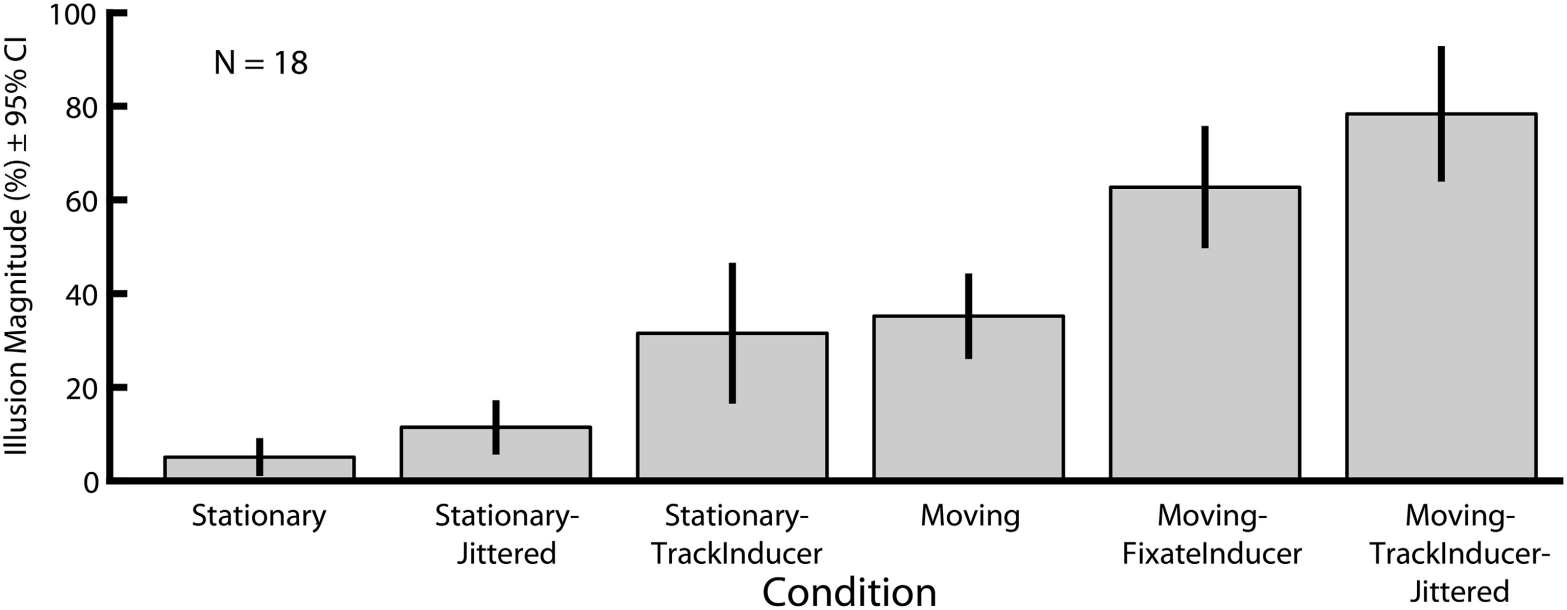
**Mean illusion magnitudes for Experiment 2**. Illusion magnitudes were significantly greater than zero for all six conditions (*p* = 0.0002 in all cases, one-sample Wicoxon signed-rank test). All pairwise comparisons, except Stationary-TrackInducer vs. Moving (*p* = 0.63, permutation test), revealed significant differences in illusion magnitudes across conditions (*p* ≤ 0.011 in all other cases). Error bars represent 95% confidence intervals.

In Experiment 2 we explored how six motion-related manipulations influence the magnitude of the Dynamic Ebbinghaus illusion (Figures 3, 4 and Movies 1–6). Figure 6 shows the mean illusion magnitudes across participants, which were significantly greater than zero for all six conditions (*p* = 0.0002 in all cases, one-sample Wilcoxon signed-rank test). These results indicate that the center circle had to be physically changing size in the same direction as the inducers for participants to perceive it as not changing over the course of the animation. Friedman's test revealed a highly significant difference among the distributions of PSEs across the six conditions [χ^2^_(5)_ = 79.30, *p* ≪ 0.00001]. Pairwise comparisons showed that the only two conditions that were not significantly different than one another were the Moving (*M* = 35.2%, *SE* = 4.0%) and Stationary-TrackInducer conditions (*M* = 31.9%, *SE* = 6.8%, *p* = 0.63, permutation test); all other pairwise comparisons revealed significant differences in the illusion magnitude across conditions (*p* ≤ 0.011 for all other comparisons, permutation test). Of particular note, the Moving (*M* = 35.2%, *SE* = 4.0%) condition led to a larger illusion magnitude than the Stationary condition (*M* = 5.1%, *SE* = 1.6%, *p* < 0.0001, permutation test), replicating the results from Experiment 1. In fact, all conditions, including the Stationary-Jittered condition (*M* = 11.4%, *SE* = 2.4%, *p* = 0.011, permutation test), which differed only in the addition of frame-by-frame jittering of the target position, led to a larger illusion magnitude than the Stationary condition. Thus, the magnitude of the Dynamic Ebbinghaus illusion was enhanced by a variety of dynamic manipulations resulting from stimulus motion or eye movements. Also of note, the illusion magnitude was stronger in the Moving-FixateInducer condition (*M* = 62.7%, *SE* = 5.8%) compared to the Stationary-TrackInducer condition (*M* = 31.9%, *SE* = 6.8%, *p* < 0.0001, permutation test), which were matched for their retinal stimulation. And finally, the Moving-TrackInducer-Jittered condition (*M* = 78.4%, *SE* = 6.5%) led to the strongest illusion of all, significantly greater than all other conditions (*p* ≤ 0.0004 for all comparisons, permutation test).

## Discussion

In the Dynamic Ebbinghaus illusion, the size and eccentricity of a set of surrounding inducers is continuously modulated between small-and-near and large-and-far. Interestingly, the results from Experiment 1 show that although this manipulation alone results in an illusory effect (~8%, Dynamic-Stationary condition), it was less than half that of the classic static Ebbinghaus (~20%, Static condition). In stark contrast, adding target motion by having the entire stimulus translate across the screen led to an illusory effect size (~37%, Dynamic-Moving condition) that was almost twice that of the classic static Ebbinghaus. It is this huge discrepancy between the Stationary and Moving dynamic conditions, also replicated in Experiment 2 (~5 vs. ~35%), that exemplifies what we have previously termed the Dynamic Illusory Size Contrast (DISC) effect (Mruczek et al., 2014). By itself, a dynamic change in the inducers is insufficient for strongly biasing perception. But when that is coupled with other dynamic motion signals, from eye movements or stimulus movements, illusory size changes are dramatic.

The Dynamic Ebbinghaus illusion shows that the DISC effect is not limited to the particular stimulus configuration we originally used to demonstrate the effect (a target bar surrounded by an expanding square box, Figure 2; Mruczek et al., 2014). Additionally, the direct comparison between the Static and Dynamic illusions (Experiment 1) shows that the subjectively strong nature of the dynamic illusion can be empirically quantified. In fact, the strongest illusory effect that we observed (Moving-TrackInducer-Jittered condition, Experiment 2) was ~78%, nearly four times the ~20% magnitude of the Static illusion observed in Experiment 1. This means that when the central circle was not physically changing size, it was perceived to shrink by over 75%. Stated another way, even if the central circle was growing to be 75% greater than its initial size, it was still perceived to be shrinking. Although some perceptual illusions can be extremely strong (e.g., Adelson, 1993; Lotto and Purves, 1999; Anderson and Winawer, 2005), the illusion magnitude observed for the Dynamic Ebbinghaus is quite dramatic compared to other illusions of size, such as the static Ebbinghaus (~10–20%, e.g., Massaro and Anderson, 1971; Jaeger and Pollack, 1977; Weintraub, 1979; Girgus and Coren, 1982; Roberts et al., 2005), Delboeuf (~7–10%, e.g., Oyama, 1962; Weintraub and Cooper, 1972; Jaeger and Lorden, 1980; Girgus and Coren, 1982; Weintraub and Schneck, 1986), Müller-Lyer (~20–40%, e.g., Dewar, 1967; Restle and Decker, 1977; Coren and Porac, 1984), and Ponzo (~10–13% for abstract version and ~40–50% for perspective version, e.g., Leibowitz and Judisch, 1967; Leibowitz et al., 1969; Girgus and Coren, 1982) illusions.

Previously, we proposed an information uncertainty hypothesis to account for the DISC effect (Mruczek et al., 2014). Perceived size is the result of the integration of multiple visual cues, including retinal image size, perceived distance, and other contextual effects such as relative size and contour interactions. Under the information uncertainty hypothesis, the contribution of each of these cues is altered or re-weighted depending on the quality of the signal it provides. Given perfect information, retinal image size and perceived distance are sufficient to construct object size, but this is often not the case. The information uncertainty hypothesis states that contextual factors will contribute more to perceived size when image size or perceived distance is noisy or uncertain. The results from the current study of the Dynamic Ebbinghaus illusion support this hypothesis. The continuous change in the size and eccentricity of the inducers in the Stationary condition (Movie 1) has little effect on perceived size because the retinal image of the target is stable over time. However, in the Moving condition (Movie 2), in which the entire stimulus translates across the screen, participants need to track the target with a smooth pursuit eye movement. Although the retinal input is largely matched for the Stationary and Moving conditions, the fact that smooth pursuit is never perfect will lead the projected image of the target to be become smeared and distorted over time. This will degrade the fidelity of any signal as to the exact size of the projected image. Under these conditions, the presence of the inducers is given more weight and has a dramatic effect on the final percept. We note that in our experiments, the difference in perceived and actual target size are likely to be driven by changes in both the relative size and separation of the target and inducers (Roberts et al., 2005). We stress, however, that the hypothesis we put forth does not assume a specific mechanism for the interactions between target and inducers, but rather specifies the conditions under which these interactions are stronger or weaker.

Experiment 2 shows that a variety of manipulations that degrade the fidelity of the image size representation over time strengthen the Dynamic Ebbinghaus illusion. Additionally, many pairwise comparisons of the conditions from Experiment 2 more specifically support the information uncertainty hypothesis. First, the most direct test of our hypothesis is the comparison of the Stationary and Stationary-Jittered conditions, which only differ by a frame-by-frame jittering of the target position. This manipulation artificially adds uncertainty to the target size. Although neither manipulation leads to a dramatic effect, adding the jitter doubled the illusion magnitude from ~5% to ~11%. Second, the Moving-FixateInducer condition led to a larger illusion than the Stationary-TrackInducer condition. These two conditions are largely matched for retinal stimulation with both involving the relative motion of the target and eyes leading to a change in target eccentricity over time, but the conditions differ in what caused the relative motion. Relative motion of the eyes and stimulus caused by movement of the eyes (Stationary-TrackInducer) could be partially accounted for in the visual system using an efference copy of the eye movement command (Bridgeman, 1995; Wurtz and Sommer, 2004). In contrast, relative motion of the eyes and stimulus caused by stimulus motion (Moving-FixateInducer) cannot be easily predicted, and thus may be expected to lead to a higher level of noise in the image size representation over time. We also observed a stronger illusory effect across matched retinal conditions when the stimulus moved compared to when the eyes moved in our previous study of the DISC effect (Mruczek et al., 2014). Third, combining the dynamic effects from eye movements, stimulus motion, and target jittering led to the largest illusion of all, ~78% (Moving-TrackInducer-Jittered condition). The partially additive effect of increasing uncertainty in the retinal input through independent manipulations is consistent with the information uncertainty hypothesis outlined above.

It is important to consider our results in the context of alternative hypotheses regarding the effects of image dynamics on the strength of the Ebbinghaus illusion. One alternative to the information uncertainty hypothesis outlined above is that image dynamics enhance the perceptual grouping of the individual stimulus components, thereby strengthening the interactions between the target and inducers. Under this hypothesis, the illusion is stronger when various cues, such as common fate motion, cause the target and inducers to be strongly grouped. Indeed, the strength of the classic Ebbinghaus illusion is modulated by target-inducer similarity (Coren and Miller, 1974; Coren and Enns, 1993; Choplin and Medin, 1999) and various perceptual grouping factors affect illusion magnitudes in other illusions, such as those related to lightness perception (Gilchrist, 2006, 2014). It is possible that factors such as common fate motion contribute to the strong illusion magnitude in some variations of the Dynamic Ebbinghaus, such as in the Moving condition compared with the Stationary condition. However, the grouping hypothesis is not a parsimonious explanation of all of our observations. Namely, it is not clear that it explains the stronger illusion magnitudes for the Stationary-Jittered and Stationary-FixInducer conditions (compared to the Stationary condition) or the Moving-FixInducer and Moving-TrackInducer-Jittered conditions (compared to the Moving condition). Across the three Stationary or the three Moving conditions there are no obvious changes in perceptual grouping factors. In contrast, the observed differences across conditions that all lack (Stationary-X) or all contain (Moving-X) the same common fate target-inducer motion are consistent with the information uncertainty hypothesis, as outlined above. Ultimately, the grouping hypothesis is empirically testable and further experiments will be needed to dissociate contributions from perceptual grouping and image size uncertainty in determining the degree to which contextual elements influence perceived size.

Another alternative to the information uncertainty hypothesis is that the illusory modulation in perceived size arises from a form of dynamic size constancy. According to this hypothesis, the expanding and contracting inducers may function as a depth cue that leads to modulations of the target's perceived depth. If the target is changing depth, but its retinal image is constant, then it must be physically changing size. Indeed, size constancy has been offered as the explanation of other illusions such as the StarTrek illusion (Qian and Petrov, 2012), the coffee cup illusion (Senders, 1966), the vista paradox (Walker et al., 1989; Reinhardt-Rutland, 1990), and the shrinking building illusion (Fukuda and Seno, 2011). As with the perceptual grouping hypothesis, although we cannot completely rule out a role for perceived distance in the Dynamic Ebbinghaus illusion, certain observations are not easy to reconcile with a purely perceived distance account. In particular, it is unclear why putative depth cues from the modulating inducers would be ineffective in the Stationary condition. If this is a special case, then why does frame-by-frame jittering of the target position in the Stationary-Jittered condition revive the illusory effect? Additionally, the size constancy account does not readily explain the additive effects of eye movements, changes in target eccentricity, and target jittering observed in Experiment 2. On the other hand, and as outlined above, these observations are readily predicted by the information uncertainty hypothesis. Lastly, the direction of size modulation that would be induced by changes in perceived depth is not entirely clear. Take, for example, the case of dynamically expanding inducers. This manipulation clearly has the potential to induce a looming percept of the inducers (i.e., the inducer appear to get closer). This could potentially cause the non-changing target to appear to recede away from the observer, as has been observed in studies of induced motion in depth by real or perceived changes in depth of a surrounding stimulus (Farne, 1972; Reinhardt-Rutland, 1983, 1984). If this were the case, then according to the principle of size-constancy the target should appear to grow in size as it recedes away. However, this is the opposite of what is observed in the Dynamic Ebbinghaus illusion and in studies of induced motion in depth (Reinhardt-Rutland, 1983, 1984, 1990); the target appeared to shrink in size as the inducers expanded. In order for the size-constancy hypothesis to account for the Dynamic Ebbinghaus illusion, the looming of the inducers must lead to a perceived looming in the non-changing target, something that is not readily observed in the demonstration videos or studies of induced motion in depth (Farne, 1972; Reinhardt-Rutland, 1983, 1984). Thus, although the size-constancy account is plausible, we believe that the information uncertainty hypothesis offers a more parsimonious explanation for the observations reported here, as well as the pattern of results reported in our previous study (Mruczek et al., 2014). However, we note that the two hypotheses need not be mutually exclusive and both depth cues and image-uncertainty may be contributing to the perceived size of the central target.

In the current experiment, we used motion-based manipulations that had a direct effect on the stability of retinal image size integrated over time. Even with perfect retinal size information, the actual size of an object cannot be unambiguously determined without a consideration of its distance from the viewer. The information uncertainty hypothesis broadly states that uncertainty in either retinal image size or perceived distance should enhance contextual influences on size perception. Uncertainty in perceived depth has been shown to alter the magnitude of classic size illusions. For example, the classic Ebbinghaus (Song et al., 2011) and the Müller-Lyer (Howard and Wagner, 1973) illusions are stronger under monocular viewing conditions, which eliminate binocular distance cues. The information uncertainty hypothesis can also explain why our stimulus does not generate (subjectively) strong changes in the perceived distance of the target, as has been reported for previous studies of induced motion in depth (Farne, 1972; Reinhardt-Rutland, 1983, 1984). Those studies utilized viewing conditions that specifically limited depth cues (e.g., monocular viewing and stimuli defined by luminesce surfaces in an otherwise completely dark room). In the case of uncertain distance cues, the information uncertainty hypothesis predicts that the perceived looming or receding of the surrounding stimulus elements may have a strong contextual influence on the perceived distance of the target. In contrast, binocular viewing and ambient light from the monitor provided strong cues to the actual distance of the target (on the stationary monitor) in our setup. By the information uncertainty hypothesis, the ability of the inducers to cause changes in the perceived depth of the target is limited in this case. However, although not sufficient to induce dramatic changes in perceived depth, it is possible that perceived looming and receding of the inducers is sufficient to reduce the overall certainty of the target's depth and as such increase the weight of the contextual influences on perceived size. As hinted above, this would be one hypothetical example of how image-uncertainty and perceived depth cues could work in tandem to influence perceived size.

Overall, the Dynamic Ebbinghaus highlights a previously underappreciated factor in size perception. Namely, stimulus dynamics can have a dramatic effect on the ability of contextual information to bias the perceived size of an object. Recently, there has been a renewed interest in understanding the neural mechanisms that support size perception. In particular, a series of neuroanatomical and behavioral studies from multiple labs point to a role for primary visual cortex in the representation of object size (Murray et al., 2006; Fang et al., 2008; Schwarzkopf et al., 2011; Song et al., 2011, 2013; Sperandio et al., 2012; Schwarzkopf and Rees, 2013). However, how representations of perceived size are neuronally instantiated and the mechanisms by which they are formed (i.e., lateral connectivity, cortical magnification, or feed-back from high visual areas) remain largely unknown. Our phenomenological results place constraints on neural and computational models implementing contextual influences on size perception. Specifically, contextual information is not integrated with representations of image size and perceived distance automatically, but rather is subject to reweighting that potentially depends on the quality of numerous cues for determining the actual size of a viewed object. Ultimately, neuroscientific approaches guided by further behavioral studies that take these factors into consideration will be required to fully account for how human size perception is achieved.

### Conflict of interest statement

The authors declare that the research was conducted in the absence of any commercial or financial relationships that could be construed as a potential conflict of interest.
