# Supplementary material for: The Dynamic Ebbinghaus: motion dynamics greatly enhance the classic contextual size illusion
Source: Front Hum Neurosci. 2015 Feb 18;9:77. doi: 10.3389/fnhum.2015.00077 (PMC4332331; doi:10.3389/fnhum.2015.00077)
Supplement: Supplementary file 1 [file DataSheet1.PDF]

Supplementary Material

The Dynamic Ebbinghaus: image size uncertainty caused by motion dynamics greatly enhances the classic contextual size illusion

Ryan E.B. Mruczek[1]*, Christopher D. Blair[1], Lars Strother[1], Gideon P. Caplovitz[1]

[1]Department of Psychology, University of Nevada Reno, Reno, NV, USA

*** Correspondence:** Dr. Ryan E.B. Mruczek, University of Nevada, Department of Psychology, 1664 North Virginia Street, Reno, NV, 89557-0296, USA.
rmruczek@unr.edu

1. Supplementary Material

In the main text, we report the statistical results derived from standard non-parametric tests and randomization procedures. Here, we briefly note the complementary statistical results derived from parametric alternatives, which in all cases led to qualitatively equivalent interpretations. All tests are two-tailed and assessed using an α of 0.05.

Experiment 1: Mean illusion magnitudes across participants were significantly greater than zero for the Static and Dynamic conditions ($p < 0.0001$ in both cases, one-sample *t*-test), and marginally significantly greater than zero for the Stationary condition ($p = 0.053$, one-sample *t*-test). A repeated-measures ANOVA revealed a highly significant difference in illusion magnitudes across the three conditions ($F_{(2,22)} = 23.0$, $p << 0.001$). Pairwise comparisons revealed that illusion magnitudes for the Dynamic-Moving condition were larger than both the Static ($t_{(11)} = 19.93$, $p = 0.005$, paired t-test) and Dynamic-Stationary ($t_{(11)} = 37.30$, $p < 0.0001$, paired t-test) conditions. Additionally, illusion magnitudes for the Dynamic-Stationary condition were significantly lower than those for the Static condition ($t_{(11)} = 8.09$, $p = 0.02$, paired t-test).

Experiment 2: Mean illusion magnitudes across participants were significantly greater than zero for all six conditions ($p < 0.005$ in all cases, one-sample *t*-test). A repeated-measures ANOVA revealed a highly significant difference in illusion magnitudes across the six conditions ($F_{(5,85)} = 53.77$, $p << 0.001$). Pairwise comparisons showed that the only two conditions that were not significantly different than one another were the Moving and Stationary-TrackInducer conditions ($t_{(17)} = 0.50$, $p = 0.62$, paired t-test); all other pairwise comparisons revealed significant differences in the illusion magnitude across conditions ($t_{(17)} > 2.33$, $p < 0.032$ in all cases, paired t-test).

2. Supplementary Figure

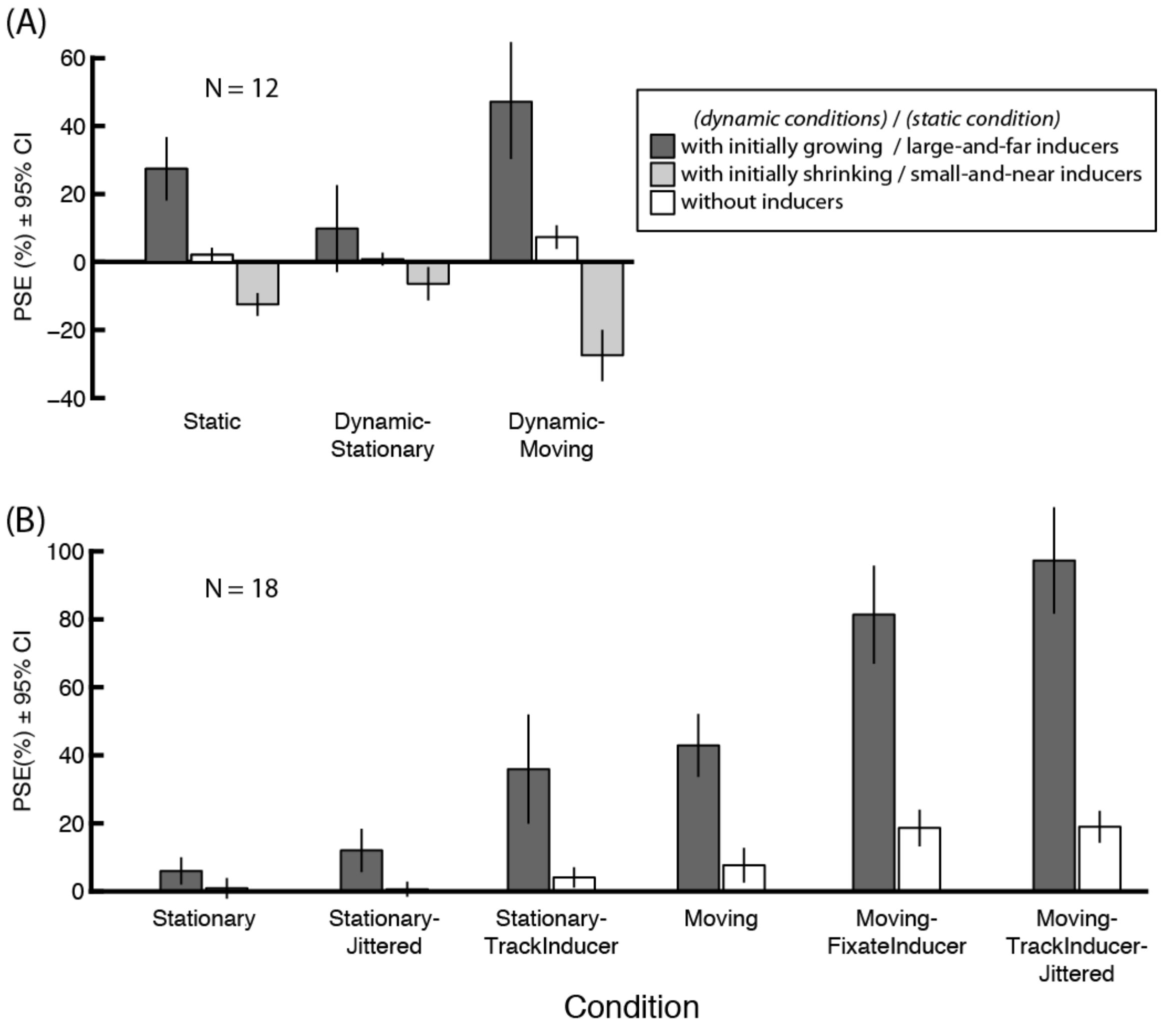

Supplementary Figure 1: Mean PSEs for the with- and without-inducer conditions for Experiments 1 and 2. The without-inducer trials (white bars) provide an opportunity to measure and account for any response bias for individual participants or perceived changes in target size that were driven by the stimulus dynamics of the target circle and the eyes alone. **(A)** Results from Experiment 1. PSEs for the without-inducer trials were significantly greater than zero for the Static (M = 2.14%, SE = 0.77%, p = 0.021, one-sample Wilcoxon signed-rank test; p = 0.018, one-sample t-test) and Dynamic-Moving (M = 7.35%, SE = 1.45%, p = 0.0005, one-sample Wilcoxon signed-rank test; p = 0.0004, one-sample t-test) conditions, but not the Dynamic-Stationary condition (M = 0.90%, SE = 0.72%, p = 0.18, one-sample Wilcoxon signed-rank test; p = 0.24, one-sample t-test). Participants adjusted the target to be slightly larger than necessary (Static condition) or to slightly grow over the first half of the animation cycle (Dynamic-Moving condition). This bias may reflect a tendency to perceive objects that are lower in the world as being closer (Roelofs and Zeeman, 1957; Sonoda, 1961; Dunn and Gray, 1965), since vertical trajectories or different vertical positions in a 3D world would typically be correlated with changes in depth. PSEs derived from each condition with inducers (gray bars) shown separately for trials in which the target was surrounded by large-and-far or small-and-near or inducers. For the dynamic conditions, this represents the trials in which the inducers initially grew or shrank, respectively. For all conditions, PSEs for the with-inducers trials differed from PSEs for the corresponding without-inducer trials. Additionally, the direction of the illusory

size change, indicated by the sign of the PSE, was expectedly inverted for trials in which the target was surrounded by large-and-far or small-and-near or inducers. The illusion magnitudes depicted in Figure 5 were calculated as the difference between the PSE for trials with and without inducers, collapsed across trials with different inducer sizes surrounding the adjustable target by averaging the absolute value of this difference in the two cases. Thus, the final illusion magnitudes reported in the main text effectively isolate the illusory contribution from the inducers by removing any effects of the target dynamics in isolation (without-inducer trials). **(B)** Results from Experiment 2. PSEs for the without-inducer trials were significantly greater than zero for the Moving (M = 7.6%, SE = 2.26%, p = 0.0007, one-sample Wilcoxon signed-rank test; p = 0.0034, one-sample t-test), but not the Stationary condition (M = 0.90%, SE = 1.08%, p = 0.71, one-sample Wilcoxon signed-rank test; p = 0.42, one-sample t-test), replicating the results from Experiment 1. For the conditions that were unique to Experiment 2, PSEs for the without-inducer trials were not significantly greater than zero for the Stationary-Jittered condition (M = 0.58%, SE = 0.69%, p = 0.78, one-sample Wilcoxon signed-rank test; p = 0.41, one-sample t-test). However, significant without-inducer PSEs were observed for the Stationary-TrackInducer (M = 3.6%, SE = 1.27%, p = 0.006, one-sample Wilcoxon signed-rank test; p = 0.11, one-sample t-test), Moving-FixateInducer (M = 18.4%, SE = 2.40%, p = 0.0002, one-sample Wilcoxon signed-rank test; $p < 0.0001$, one-sample t-test), and Moving-TrackInducer-Jittered (M = 18.9%, SE = 2.09%, p = 0.0002, one-sample Wilcoxon signed-rank test; $p < 0.0001$, one-sample t-test) conditions. For these three conditions, the eccentricity of the peripherally viewed target changed over time. As such, the observed biases are consistent with previous studies reporting changes in perceived size with retinal eccentricity (Helmholtz, 1867; James, 1890; Bedell and Johnson, 1984). As with Experiment 1, we accounted for any biases in perceived size apparent in the without-inducer trials by subtracting the without-inducer PSE from the with-inducer PSE for a given condition. The resulting illusion magnitudes for Experiment 2 are depicted in Figure 6.

3. Supplementary Movie Legends

Movie 1: In the Stationary condition (Experiments 1 and 2), the participant fixated the green dot on the central target circle while the inducers continuously changed size and eccentricity between small-and-near and large-and-far.

Movie 2: In the Moving condition (Experiments 1 and 2), the participant tracked the green dot on the central target circle while the inducers continuously changed size and eccentricity and the entire stimulus translated across the screen. For Experiment 2, the angle of translation was altered slightly so that the upper-left inducer did not change position during the animation.

Movie 3: In the Stationary-Jittered condition (Experiment 2), the participant fixated the green dot on the central target circle while the inducers continuously changed size and eccentricity. The exact position of the target was jittered by a small amount on a frame-by-frame basis.

Movie 4: In the Stationary-TrackInducer condition (Experiment 2), the participant tracked the green dot on the upper-left inducer while the inducers continuously changed size and eccentricity.

Movie 5: In the Moving-FixateInducer condition (Experiment 2), the participant fixated the green dot on the upper-left inducer while the inducers continuously changed size and eccentricity and the entire stimulus translated across the screen.

Movie 6: In the Moving condition (Experiment 2), the participant tracked the green dot on the upper-right inducer while the inducers continuously changed size and eccentricity and the entire stimulus translated across the screen. The exact position of the target was jittered by a small amount on a frame-by-frame basis.

4. References

Bedell, H.E., and Johnson, C.A. (1984). The perceived size of targets in the peripheral and central visual fields. *Ophthalmic and Physiological Optics* 4**,** 123-131.

Dunn, B.E., and Gray, G.C. (1965). Relative height on the picture-plane and depth perception. *Percept Mot Skills* 21**,** 227-236.

Helmholtz, H.V. (1867). *Handbuch der Physiologischen Optik (1st ed.).* Leipzig: Voss.

James, W. (1890). *Principles of Psychology.* London: Macmillan.

Roelofs, C.O., and Zeeman, W.P. (1957). Apparent size and apparent distance in binocular and monocular vision. *Ophthalmologica* 133**,** 188-204.

Sonoda, G. (1961). Perceptual constancies observed in plane pictures. *Experimental researches on the structure of the perceptual space* 4**,** 199-228.
